# Supplementary material for: Performance of Screening Strategies for Latent Tuberculosis Infection in Patients with Inflammatory Bowel Disease: Results from the ENEIDA Registry of GETECCU
Source: J Clin Med. 2022 Jul 5;11(13):3915. doi: 10.3390/jcm11133915 (PMC9267853; doi:10.3390/jcm11133915)
Supplement: Supplementary file 1 [file jcm-11-03915-s001.zip › jcm-1765393-supplementary.pdf]

Supplementary Materials. List of centres participating in the study;

. Hospital Universitario Central de Asturias, and Instituto de Investigación Sanitaria del Principado de Asturias (ISPA), Oviedo, Spain
. Hospital Clínico Universitario San Carlos, and Instituto de Investigación del Hospital Clínico San Carlos [IdISSC], Madrid, Spain
. Hospital Universitari Mútua Terrassa, Centro de Investigación Biomédica en Red de Enfermedades Hepáticas y Digestivas (CIBERehd), Terrassa, Spain
. Complexo Hospitalario Universitario de Pontevedra, Pontevedra, Spain
. Hospital Universitario de La Princesa, Instituto de Investigación Sanitaria Princesa (IIS-IP), Universidad Autónoma de Madrid (UAM), Centro de Investigación Biomédica en Red de Enfermedades Hepáticas y Digestivas (CIBERehd), Madrid, Spain
. Hospital Universitario Río Hortega, Valladolid, Spain
. Hospital Universitario Marqués de Valdecilla, Universidad de Cantabria, Instituto de Investigación Sanitaria Valdecilla (IDIVAL), Santander, Spain
. Hospital Universitario Ramón y Cajal, Madrid, Spain
. Hospital Universitari i Politecnic La Fe and CIBERehd, Valencia, Spain
. Hospital Universitario Miguel Servet, Instituto de Investigación Sanitaria Aragón (IISA), Zaragoza, Spain
. Hospital Universitari Germans Trials i Pujol and CIBERehd, Badalona, Spain
. Hospital Universitario La Paz, and Instituto de Investigación Sanitaria del Hospital La Paz (IdiPAZ), Madrid, Spain
. Hospital Universitario Fundación Alcorcón, Madrid, Spain
. Hospital Universitario de Bellvitge, and Institut d'Investigació Biomèdica de Bellvitge (IDIBELL), Barcelona, Spain
. Hospital Universitario de Salamanca, Salamanca, Spain
. Hospital Nuestra Señora de la Candelaria, Tenerife, Spain
. Hospital de Galdakao-Usansolo, Galdakao, Vizcaya, Spain
. Hospital General Universitario de Elche, Alicante, Spain
. Hospital Sant Joan Despí-Moisès Broggi, Barcelona, Spain
. Hospital San Jorge, Huesca, Spain
. Hospital Clinic i Provincial and CIBERehd, IDIBAPS, Barcelona, Spain
. Hospital Universitario de Fuenlabrada and Instituto de Investigación Sanitaria . Hospital La Paz (IdiPaz), Madrid, Spain
. Hospital Universitari Parc Taulí, Sabadell, Depàrtament de Medicina, Universitat Autònoma de Barcelona and CIBERehd, Barcelona, Spain
. Consorcio Sanitario de Terrasa, Barcelona, Spain
. Hospital de la Santa Creu i Sant Pau, Barcelona, Spain
. Hospital del Mar and Institut Hospital del Mar d'Investigacions Mèdiques, Barcelona, Spain
. Hospital Clínico Universitario de Valencia, Valencia, Spain
. Hospital Universitario de Torrejón, Madrid, Spain
. Hospital Universitario de Donostia/Biodonostia, Universidad del País Vasco (UPV/EHU), CIBERehd, San Sebastián, Spain
. Hospital Universitario de Girona Dr Josep Trueta, Girona, Spain
. Hospital Universitario de Burgos, Burgos, Spain
. Hospital Reina Sofía, IMIBIC, Córdoba, Spain

. Hospital Clínico de Málaga, Málaga, Spain

Table S1. Factors associated with diagnosis of LTBI in IBD patients who were not undergoing anti-TNF treatment at screening

	Univariable analysis			Multivariable analysis		
	Odds ratio	95% CI	p	Odds ratio	95% CI	p
Gender						
Female	Reference			Reference		
Male	1.21	1.07-1.36	0.002	1.20	1.06-1.36	0.003
Age at TB screening, years	1.04	1.03-1.04.	<0.001	1.04	1.04–1.05	<0.001
Type of IBD						
IBD-unclassified	Reference			Reference		
Ulcerative colitis	0.84	0.57-1.27	0.40	0.78	0.52–1.21	0.25
Crohn´s disease	0.90	0.62-1.36	0.62	0.92	0.61–1.41	0.70
Smoking status						
Never	Reference			Reference		
Ever	1.58	1.40-1.79	<0.001	1.53	1.34–1.75	<0.001
Unknown	1.03	0.79-1.32	0.81	0.86	0.65–1.13	0.29
IMM at TB screening						
No	Reference			Reference		
Yes	0.82	0.72-0.94	0.005	0.82	0.71–0.95	0.007
Geographical area						
Madrid	Reference			Reference		
Aragón	0.87	0.67-1.12	0.28	0.91	0.69–1.19	0.50
Asturias	0.98	0.74-1.29	0.90	1.16	0.87–1.54	0.30
Canarias	0.70	0.40-1.15	0.18	0.59	0.33–1.01	0.07
Cantabria	1.34	1.07-1.68	0.009	1.39	1.10–1.75	0.006
Castilla-León	1.52	1.25-1.84	<0.001	1.99	1.61–2.45	<0.001
Catalonia	1.25	1.06-1,46	0.007	1.47	1.25–1.75	<0.001
Basque country	0.67	0.44-10.98	0.05	0.79	0.52–1.17	0.26
Valencia	0.55	0.42-0.71	<0.001	0.63	0.47-0.82	0.001

TB incidence in country of origin						
Low / intermediate	Reference			Reference		
High	2.13	1.64-2.75	<0.001	3.53	2.68–4.64	<0.001
Year of TB screening	0.97	0.96-0.99	0.003	0.93	0.91–0.95	<0.001

CI: confidence interval; TB tuberculosis; IBD: inflammatory bowel disease; IMM: immunomodulator;

Table S2. Frequency of LTBI among adult patients with IBD according to age and gender

		Patients with LTBI, number (%)		
**age**	**Number of patients (M/F)**	**Male**	**Female**	**P value**
**<26**	1136 (642/494)	45 (7·0)	34 (6·9)	1
**26–35**	1621 (832/789)	91 (10·9)	92 (12)	0·70
**36–45**	1819 (925/894)	166 (18)	147 (16)	0·43
**46–55**	1466 (762/704)	218 (29)	183 (26)	0·29
**56–65**	958 (529/429)	190 (36)	100 (23)	<0·001
**>65**	594 (298/296)	106 (36)	73 (25)	0·005
**Overall**	7594 (3988/3606)	816 (21)	629 (17)	0·01

LTBI: latent tuberculosis infection; M: male; F: female

Figure S1.- Frequency of LTBI, positive result in TST and IGRA, depending on the year in which screening was performed in Spanish patients with IBD.

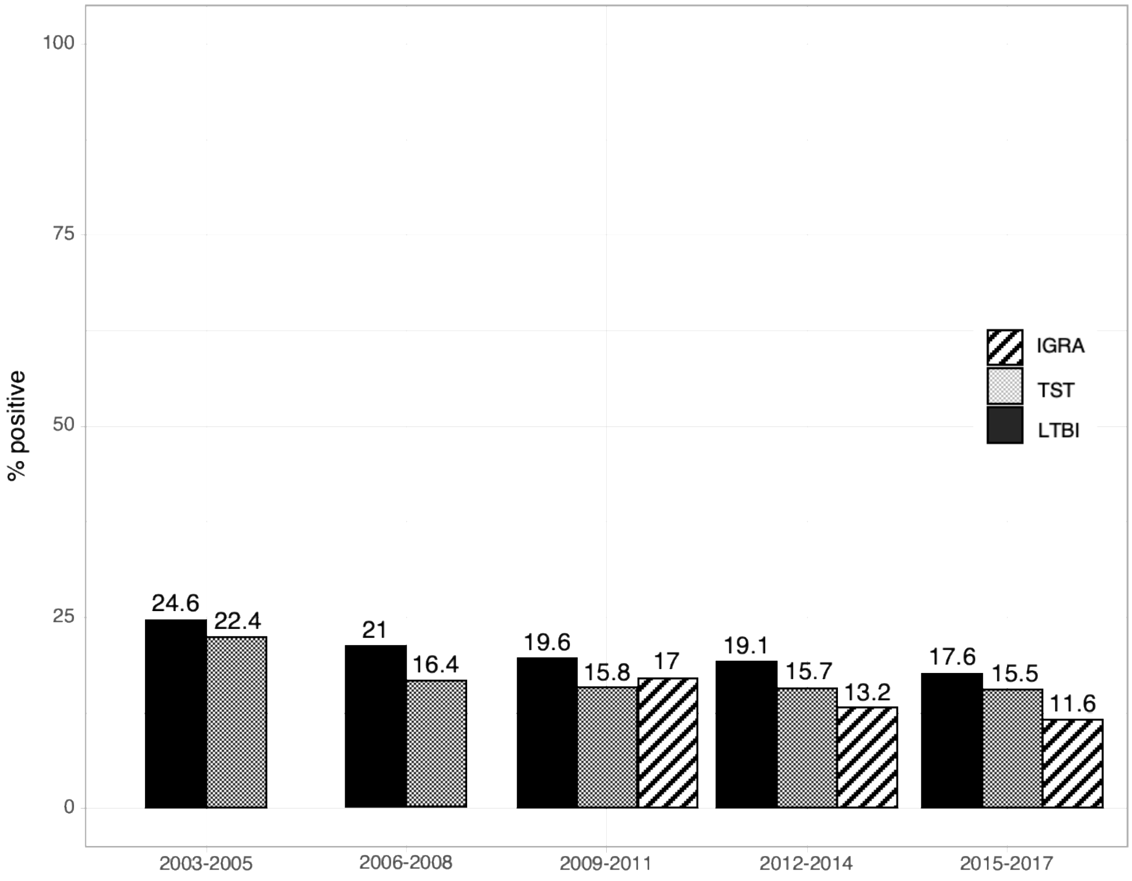

_LTBI: latent tuberculosis infection; TST: tuberculin skin test; IGRA: interferon gamma release assay

Table S3. Probability (%) of LTBI in IBD patients who were not undergoing anti-TNF treatment at screening according to gender, smoking status, immunomodulator use at screening, strategy of screening (dual versus single, early versus routine) and origin in a country with a high or intermediate/low incidence of TB.

			Men						Women					
			IGRA and TST		IGRA		TST		IGRA and TST		IGRA		TST	
			No IMM	With IMM	No IMM	With IMM	No IMM	With IMM	No IMM	With IMM	No IMM	With IMM	No IMM	With IMM
Early screening	High TB incidence	Ever smoked	53	51	40	38	46	43	49	46	36	34	41	39
		Never smoked	41	39	29	27	34	32	37	35	26	24	30	28
	Low/intermediate TB incidence	Ever smoked	31	29	21	20	25	24	28	26	19	17	22	21
		Never smoked	22	20	14	13	17	16	19	18	12	11	15	14
Routine screening	High TB incidence	Ever smoked	43	41	31	29	36	34	39	37	28	26	32	30
		Never smoked	32	30	22	20	26	24	28	27	19	18	23	21
	Low/intermediate TB incidence	Ever smoked	24	22	16	14	19	17	21	19	14	12	16	15
		Never smoked	16	15	10	9	12	11	14	13	9	8	11	10

IGRA: interferon gamma release assay; TST: tuberculin skin test; IMM: immunomodulator; TB: tuberculosis
